# Perforation of the descending colon induced by alectinib in a patient with non-small cell lung cancer: a Case Report

**DOI:** 10.3389/fphar.2025.1545614

**Published:** 2025-05-21

**Authors:** Yun Li, Ping Yu, Ran Wang

**Affiliations:** Department of Pharmacy, Nanjing Lishui People's Hospitial (Zhongda Hospital Lishui Branch), Southeast University, Nanjing, China

**Keywords:** ALK, non-small cell lung cancer, alectinib, adverse event, perforation of the descending colon

## Abstract

Anaplastic lymphoma kinase (ALK) inhibitor alectinib has demonstrated significant potential in treating non-small cell lung cancer (NSCLC); however, its adverse effects remain a notable challenge for healthcare professionals. This report examines the case of a 79-year-old female lung cancer patient who presented with persistent colic in the left abdomen 10 months after initiating alectinib treatment. Upon admission, abdominal computed tomography revealed mild thickening of the descending colon wall, accompanied by free gas and surrounding exudation, leading to a diagnosis of descending colon perforation. After excluding alternative causes of gastrointestinal perforation, the condition was attributed to a severe adverse reaction associated with alectinib. While alectinib has been reported to cause serious gastrointestinal perforations, the underlying mechanism remains unclear and warrants further clinical investigation. Clinicians should be vigilant in recognizing and promptly managing this potential complication during alectinib therapy.

## Introduction

Alectinib, a second-generation anaplastic lymphoma kinase (ALK) inhibitor, has demonstrated proven efficacy and a favorable tolerability profile, making it a recommended first-line therapy for patients with ALK-positive NSCLC (National Comprehensive Cancer Network and Inc, 2022). Despite its widespread clinical use, reports of adverse reactions have emerged, most commonly including rash, liver dysfunction, hemolytic anemia, and interstitial lung disease (Alecensa@ package insert, 2021). However, gastrointestinal perforation associated with ALK inhibitors is a rare but potentially life-threatening complication. This report presents a case of an ALK-positive NSCLC patient who developed descending colon perforation following alectinib administration.

## Case presentation

**FIGURE 1:**
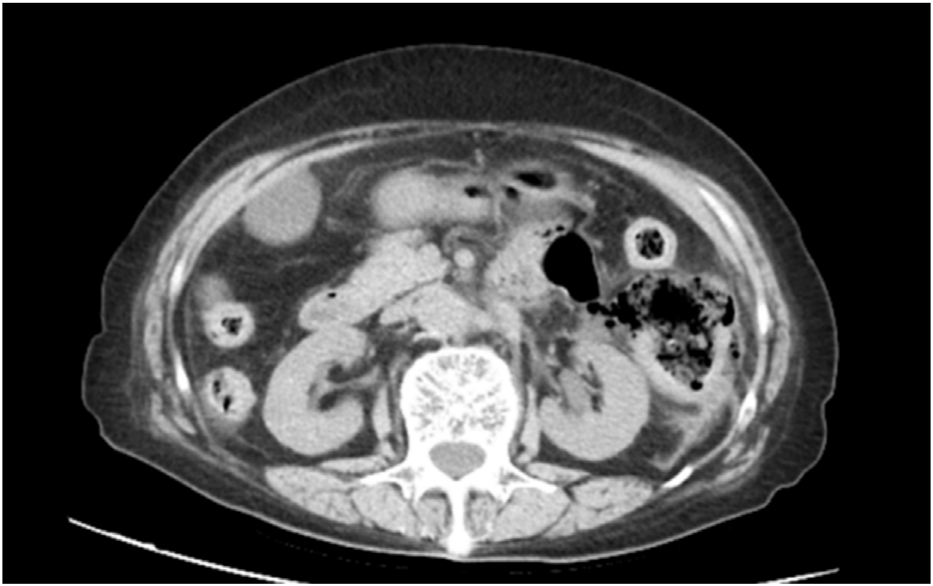
Abdominal computed tomography (CT) revealed mild thickening of the descending colon wall accompanied by free gas and surrounding exudation, a perforation of the digestive tract was considered.

A 79-year-old female lung cancer patient presented with persistent abdominal colic lasting 7 hours. Her physical examination findings were as follows: body temperature of 37.7°C, heart rate of 98 beats per minute, and blood pressure (BP) of 117/90 mmHg. The blood test results were as follows: white blood cell (WBC) count of 9.3 × 10^9^/L (normal: 4.0–10.0 × 10^9^/L); neutrophil (NE) count of 8.16 × 10^9^/L (normal: 2.0–7.0 × 10^9^/L); and C-reactive protein (CRP) level of 36.03 mg/L (normal: 0–10 mg/L). Her liver and kidney functions were normal. Whole-abdominal CT imaging indicated a possible perforation in the digestive tract (Figure 1). During laparotomy, a 5.0 cm perforation was identified in the descending colon near the splenic flexure; no palpable tumor or lymphadenopathy was observed. Surgical intervention involved partial resection of the descending colon with transverse colostomy, and specimens from the resected colon and greater omentum were collected for pathological analysis.

The patient was diagnosed with lung cancer 10 months prior to admission and had been receiving alectinib treatment for approximately 10 months. Since her NSCLC diagnosis, she had not been treated with any chemotherapy or taken any medications or supplements other than alectinib. In the early stage of treatment, the patient occasionally experienced nausea approximately 30 min after taking the medication, accompanied by mild abdominal pain from time to time. The symptoms alleviated after about 20 min of rest. The patient considered these discomforts as common gastrointestinal adverse reactions of alectinib. About 2 months later, the patient experienced discomfort from constipation, having bowel movements only once or twice a week. At this time, the patient needed to use glycerin enemas to defecate normally. As the treatment progressed, the patient’s constipation became increasingly severe. Even after using glycerin enemas, satisfactory results were not achieved, and the patient also developed frequent abdominal pain and discomfort. The patient has no previous history of gastrointestinal diseases, family medical history of colon perforation, or drug allergy history. Moreover, before this hospitalization, the patient had not experienced factors that could cause intestinal perforation, such as abdominal trauma, bacterial gastroenteritis, or intestinal surgery.

**FIGURE 2:**
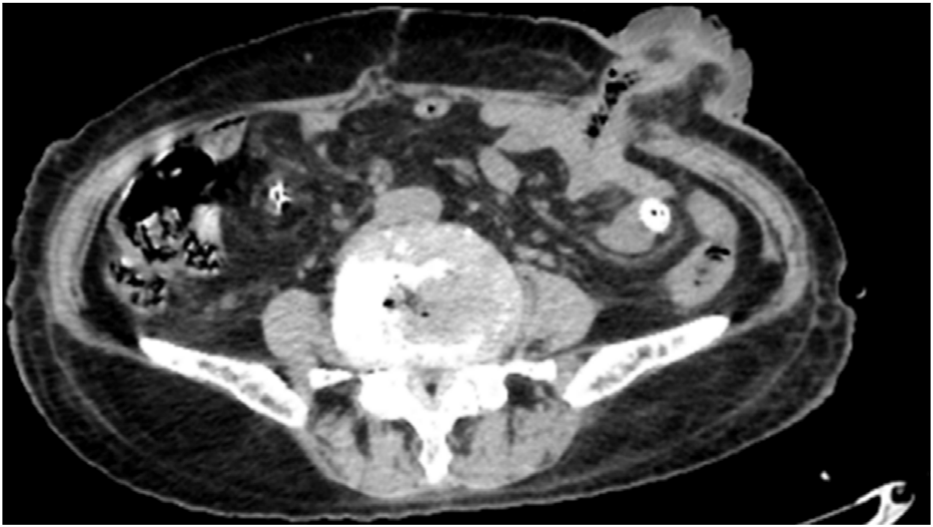
The patient’s bowel was open and no obstruction was found.

**FIGURE 3:**
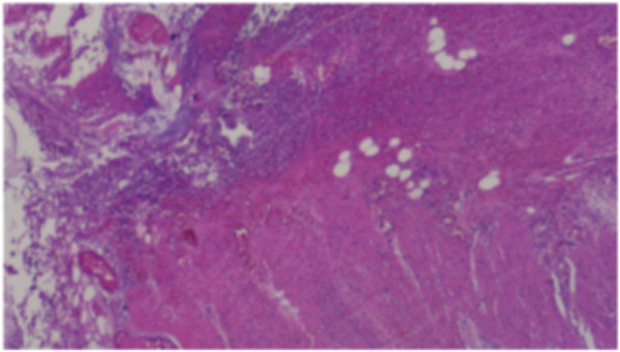
Postoperative pathological analysis. (Partial colon): A ruptured area measuring approximately 2.5 cm in maximum diameter was identified in the colon wall. Histopathological examination revealed full-thickness necrosis of the intestinal wall accompanied by extensive infiltration of acute and chronic inflammatory cells within the surrounding adipose tissue, along with evidence of congestion and hemorrhage. These findings aligned with clinical indications of colon perforation. The resected margins at both ends of the intestinal wall exhibited no significant pathological abnormalities. (Greater omentum): The omental tissue displayed localized vascular proliferation, along with small infiltration of acute and chronic inflammatory cells. No other pathological abnormalities were detected.

Following surgery, the patient underwent oxygen therapy, gastric tube insertion, empiric antimicrobial therapy with piperacillin-tazobactam, gastric acid suppression, and mucosal protection with rabeprazole, as well as parenteral nutritional support and pain management. *Helicobacter pylori* testing yielded a negative result. A repeat abdominal CT scan on postoperative day 7 (Figure 2) demonstrated unobstructed intestinal tracts and normal stoma function, as evidenced by effective excretion (Figure 3).

Upon clinical stabilization, referral to the oncology department for adjustment of the anti-tumor regimen was recommended. However, the patient opted to continue alectinib therapy for lung cancer instead.

## Discussion

To the best of our knowledge, this case represents the first documented instance of gastrointestinal perforation associated with alectinib, an exceedingly rare and potentially life-threatening adverse effect. A comprehensive search of the PubMed, Web of Science, and Embase databases was conducted using the terms “alectinib,” “case report,” “adverse,” “induced,” and “related.” The analysis of the retrieved cases indicated that alectinib-related adverse drug reactions (ADRs) predominantly affected the skin and its appendages, followed by the respiratory, urinary, cardiovascular, and hematologic systems (Seegobin et al., 2020; Gleue et al., 2021; Farooq et al., 2020; Myall et al., 2021; Gadotti et al., 2021; Ramachandran et al., 2018; Nagai et al., 2018; Shimada et al., 2017; Okumoto et al., 2021; Yuan et al., 2020; Rao et al., 2020). Notably, no prior reports of gastrointestinal perforation associated with alectinib were identified.

Alectinib, a second-generation ALK inhibitor, is widely recognized as a first-line therapeutic agent for ALK-positive NSCLC. The U.S. Food and Drug Administration (FDA) granted its approval for first-line use in ALK-positive NSCLC patients in November 2017, followed by the National Medical Products Administration (NMPA)’s authorization in China in August 2018 for the management of locally advanced or metastatic ALK-positive NSCLC. Treatment in this case report was initiated following confirmation of ALK positivity through genetic testing.

Gastrointestinal (GI) perforation is commonly associated with conventional cytotoxic chemotherapy and metastatic tumors. However, GI metastasis from lung cancer is rare. Kim et al. (Kim et al., 2009) reported that GI metastasis occurred in only 0.19% of patients with lung cancer. Although GI metastasis from lung cancer is typically characterized by acute abdominal symptoms and a fulminant course, biopsy and pathological analysis of the patient’s colon and greater omentum (Figure 3) confirmed the absence of gastrointestinal metastasis. Previous literature includes two cases of gastrointestinal perforation linked to epidermal growth factor receptor (EGFR) tyrosine kinase inhibitors (TKIs) in the absence of gastrointestinal metastasis (Gass-Jégu et al., 2016). Given the shared pharmacodynamic characteristics of ALK inhibitors, including their rapid action, similar outcomes have been observed. For instance, a reported case of crizotinib-related gastrointestinal perforation (YANAGISAWA et al., 2017) suggests that such adverse effects associated with molecular targeted therapies may arise independently of tumor necrosis.

Alectinib-induced gastrointestinal perforation may be correlated with its plasma concentration. The standard recommended dose of alectinib is 600 mg twice daily (bid) in China, Europe, and North America, with the frequency and severity of adverse reactions in the package insert based on this dosage. Evidence suggests that reducing the dose to 300 mg bid significantly decreases both the incidence and severity of adverse drug reactions (ADRs) compared to the standard 600 mg bid regimen (DBSA and JAL, 2021). In this case, the patient received 600 mg bid. Notably, alectinib is primarily eliminated via the hepatobiliary pathway (98%), potentially exposing gastrointestinal cells or vasculature to elevated drug concentrations, thereby inducing toxicity. Additional risk factors for lower gastrointestinal perforation include age-related intestinal wall fragility, NSAID-induced mucosal damage, and constipation-related increased intraluminal pressure. While *H. pylori* is a known risk factor for upper gastrointestinal perforation, its role in colonic perforation remains unclear.

Following the exclusion of other risk factors, an adverse drug reaction (ADR) score of nine was determined using the Naranjo assessment scale, categorizing the correlation between the patient’s descending colon perforation and alectinib as “definite.” The gastrointestinal perforation may be attributed to the following mechanisms: (1) alectinib’s direct cytotoxicity on gastrointestinal cells, leading to necrosis of intestinal wall cells and disruption of microvascular circulation; (2) alectinib-induced constipation, resulting in increased intestinal content and pressure. These effects, compounded by age-related fragility and degeneration of the intestinal wall in elderly patients, ultimately led to the severe complication of gastrointestinal perforation.

In clinical practice, the close monitoring of potential adverse reactions to alectinib is essential, particularly given the risk of rare but life-threatening complications such as gastrointestinal perforation, ulcer formation, and severe constipation. If patients present with persistent abdominal pain, altered bowel habits, or bloating, an abdominal CT scan or ultrasound should be promptly performed to evaluate bowel wall thickness and detect the presence of free air in the abdominal cavity. This assessment should be supplemented with inflammatory markers, such as C-reactive protein (CRP) and white blood cell (WBC) counts, to determine the severity of the condition (Gass-Jégu et al., 2016; YANAGISAWA et al., 2017).

Moreover, appropriate dose management plays a pivotal role in mitigating the risk of severe adverse events. Previous studies have demonstrated that reducing the alectinib dosage from 600 mg twice daily to 300 mg twice daily can significantly decrease both the incidence and severity of adverse reactions (DBSA and JAL, 2021). Consequently, in older adults or individuals with a history of gastrointestinal diseases (e.g., chronic inflammation or prior gastrointestinal surgery), a more frequent and comprehensive follow-up strategy—incorporating regular imaging evaluations, laboratory tests, and symptom assessments—should be implemented early in the treatment course, ensuring timely intervention if warning signs arise.

By adopting individualized dose adjustments and rigorous monitoring protocols, clinicians can effectively minimize severe complications while maintaining antitumor efficacy. These strategies not only offer crucial guidance for subsequent treatment decisions, such as switching to an alternative ALK-TKI, but also contribute to enhancing overall treatment safety and improving patient survival outcomes.

## Conclusion

Non-small cell lung cancer (NSCLC) is a formidable disease that remains challenging to treat. The discovery of ALK-TKI inhibitors has brought new hope for improving patient survival rates. However, alongside these advancements, clinicians face the challenge of identifying and managing increasingly complex adverse effects. The mechanism underlying alectinib-induced gastrointestinal perforation remains unclear and warrants further clinical investigation. Clinicians should develop a deeper understanding of potential side effects and implement appropriate management strategies to mitigate the risk of severe complications.

## Data Availability

The original contributions presented in the study are included in the article/supplementary material, further inquiries can be directed to the corresponding author.
